# Impact of COVID-19 on corneal donation and distribution

**DOI:** 10.1177/1120672120948746

**Published:** 2020-08-05

**Authors:** Mohit Parekh, Stefano Ferrari, Vito Romano, James Myerscough, Gary LA Jones, Carlo Griffoni, Sajjad Ahmad, Giuseppe Feltrin, Massimo Busin, Diego Ponzin

**Affiliations:** 1 Institute of Ophthalmology, University College London, London, UK; 2 Fondazione Banca degli Occhi del Veneto Onlus, Mestre, Venice, Italy; 3 St. Paul’s Eye Unit, Royal Liverpool University Hospital, Liverpool, UK; 4 Department of Ophthalmology, Southend NHS University Hospital, Southend, UK; 5 Department of Ophthalmology, Villa Igea Private Hospital, Forli, Italy; 6 Moorfields Eye Hospital NHS Trust Foundation, London, UK; 7 Regional Centre for Transplant Coordination, Padua, Italy; 8 Department of Morphology, Surgery and Experimental Medicine, University of Ferrara, Ferrara, Italy

Dear Editor,

The potential for transmission of the Severe Acute Respiratory Syndrome Coronavirus 2 (SARS-CoV-2)^1^ through human cells or tissue transplantation is still under investigation. SARS-CoV-2 has been detected in tears and conjunctival secretions suggesting that transplanted ocular tissues may carry the risk of donor-recipient transmission.^2^ As a result, stringent regulations have been issued to reduce potential contamination from donors leading to a significant decrease in the corneal procurement and distribution, which is also observed due to pandemic related cessation of elective surgeries. We report the effect of COVID-19 pandemic on the rate of donation and distribution of corneal tissues observed at Fondazione Banca degli Occhi del Veneto (FBOV – Venice, Italy), one of the largest eye banks in Europe. A two-tailed non-parametric Mann–Whitney test with 95% CI was used to check the statistical difference between the tissues procured and distributed for transplantation in 2019 and 2020. Retrospective data collected from FBOV database during the lockdown period (between 9th March and 8th May 2020) showed a significant decline in the number of tissues procured (–41%; *p* < 0.0001) and distributed for transplantation (–62%; *p* < 0.0001). However, during the first week after the lockdown was eased (11th–14th May), we observed that the donation rate did not improve significantly (–30%; *p* = 0.4578) but the tissues requested for transplantation inclined (+14%; *p* = 0.5065) soon after the elective surgeries were partially resumed.

Following the outbreak of the COVID-19 pandemic, guidelines from the societies concerning eye banks such as the Eye Bank Association of America^3^ and the Global Alliance of Eye Bank Associations (GAEBA)^4^ were released with the aim to largely exclude donor tissues positive for, or in recent close contact with, COVID-19 patient or deceased. In Italy, indications from the Ministry of Health required to perform nasopharyngeal swabs on the donors and to obtain a confirmed negative result for SARS-CoV-2 before the tissue is released for transplantation, which has now become mandatory for all the donors. The implementation of these guidelines has reduced the probability of inadvertent distribution of corneas from asymptomatic donors, which has emerged as a significant feature of COVID-19.^5^ In FBOV, of the 301 nasopharyngeal swabs performed (donors with the above inclusion criteria), only three donors were confirmed as SARS-CoV-2 positive. It is conceivable however that false negative rate encountered with PCR testing could lead to an underestimated value of truly affected donors. A key issue of COVID-19 pandemic is the cancellation of all elective surgeries. This is in part due to hospital reorganisation and redeployment of the staff from different specialties recruited to help intensive care units. Furthermore, non-imminent sight-threating surgery has been postponed to avoid ‘at-risk’ cohort of patients typically being elderly. Strict regulations, higher maintenance costs and cancellation of elective surgeries have significantly challenged the eye banks. Although COVID-19 cases are flattening over time and the lockdown is partially eased, a further delay is expected before most centres are fully operational. The safety measures implemented by the eye banks will not be relaxed in the foreseeable future, with testing for SARS-CoV-2 becoming part of routine screening practices together with HIV and HCV. Moreover, approximately 4% of donors died from respiratory related complications (FBOV data) in 2019; a group that will now have to be carefully scrutinised.

An estimated 12.7 million patients are waiting for corneal transplants worldwide^6^ and because of the pandemic this demand is expected to rise significantly which may become a serious threat to carrying out sight-saving surgery in the near future. To ease the immediate pressure on the number of available tissues, it would be reasonable to utilise a single tissue for multiple transplants either as anterior/posterior lamellar grafts or by dividing a single layer into multiple grafts.^7^ In addition, research on whether SARS-CoV-2 can be completely eliminated from an ex vivo donor cornea will be extremely necessary in order to recover the currently reduced supply and distribution of donor tissues for corneal transplants.
